# Zinc-α2-Glycoprotein Is Unrelated to Gestational Diabetes: Anthropometric and Metabolic Determinants in Pregnant Women and Their Offspring

**DOI:** 10.1371/journal.pone.0047601

**Published:** 2012-12-18

**Authors:** Silvia Näf, Xavier Escote, Rosa Elena Yañez, Mónica Ballesteros, Inmaculada Simón, Pilar Gil, Ana Megia, Joan Vendrell

**Affiliations:** 1 Endocrinology and Diabetes Unit, Hospital Universitari de Tarragona Joan XXIII, Institut d'Investigació Sanitària Pere Virgili (IISPV), Universitat Rovira i Virgili, Tarragona, Spain; 2 Centro de Investigación Biomédica en Red de Diabetes y Enfermedades Metabólicas Asociadas (CIBERDEM), Instituto de Salud Carlos III, Madrid, Spain; 3 Obstetrics and Gynecology Service, Hospital Universitari de Tarragona Joan XXIII, IISPV, Universitat Rovira i Virgili, Tarragona, Spain; University of Hyderabad, India

## Abstract

**Context:**

Zinc-α2-Glycoprotein (ZAG) is an adipokine with lipolytic action and is positively associated with adiponectin in adipose tissue. We hypothesize that ZAG may be related with hydrocarbonate metabolism disturbances observed in gestational diabetes mellitus (GDM).

**Objective:**

The aim of this study was to analyze serum ZAG concentration and its relationship with carbohydrate metabolism in pregnant women and its influence on fetal growth.

**Design:**

207 pregnant women (130 with normal glucose tolerance (NGT) and 77 with GDM) recruited in the early third trimester and their offspring were studied. Cord blood was obtained at delivery and neonatal anthropometry was assessed in the first 48 hours. ZAG was determined in maternal serum and cord blood.

**Results:**

ZAG concentration was lower in cord blood than in maternal serum, but similar concentration was observed in NGT and GDM pregnant women. Also similar levels were found between offspring of NGT and GDM women. In the bivariate analysis, maternal ZAG (mZAG) was positively correlated with adiponectin and HDL cholesterol, and negatively correlated with insulin and triglyceride concentrations, and HOMA index. On the other hand, cord blood ZAG (cbZAG) was positively correlated with fat-free mass, birth weight and gestational age at delivery. After adjusting for confounding variables, gestational age at delivery and HDL cholesterol emerged as the sole determinants of cord blood ZAG and maternal ZAG concentrations, respectively.

**Conclusion:**

mZAG was not associated with glucose metabolism during pregnancy. ZAG concentration was lower in cord blood compared with maternal serum. cbZAG was independently correlated with gestational age at delivery, suggesting a role during the accelerated fetal growth during latter pregnancy.

## Introduction

Late pregnancy is characterized by an insulin resistance state which involves changes in lipid and glucose metabolism [1] to meet the increased metabolic demands of the fetus. The decrease in insulin sensitivity is offset by an increase of pancreatic insulin secretion, but when this mechanism is insufficient, gestational diabetes mellitus (GDM) develops.

Adipose tissue has a recognized capacity to secrete several hormones called adipokines, which modulate the action of insulin in different tissues, including adipocytes themselves [2]. In addition, some of these proteins have been involved in the regulatory process of energy homeostasis and weight control. Nowadays, there is growing interest in the role of these adipokines as contributors to the metabolic abnormalities both in the mother and the fetus, observed in the GDM context. Thus, lower levels of total adiponectin [3]–[5] and its multimeric forms [4]–[6] have been reported in GDM. More recently, up-regulation of adipocyte fatty acid-binding protein and of retinol-binding protein 4 in mothers has been related with GDM insulin-resistant condition [7].

Zinc-α2-Glycoprotein (ZAG) is a soluble protein of 41 kD identified in secretory epithelial cells from different organs such as the liver, prostate, breast, kidney and lung [8]. Recently, ZAG gene and protein expression have also been detected in human visceral and subcutaneous adipose tissues [9]. In turn, mature adipocytes may synthesize and secrete this protein suggesting a possible role in adipose tissue metabolism in the context of obesity and insulin resistance [10]. In fact, one of the main functions of ZAG in adipose tissue is thought to be its lipolytic action. ZAG has been shown to stimulate lipolysis in murine epididymal adipocytes associated with the activation of adenylate cyclase in a GTP-dependent manner [11]. This lipolytic effect seems to be mediated through the stimulation of a β3 receptor [12]. *ZAG* expression was down-regulated in subcutaneous adipose tissue (SAT) [13]–[14] and in visceral adipose tissue (VAT) from obese patients [14] suggesting that *ZAG* expression in human adipose tissue appears to be inversely linked to fat mass. Besides, *ZAG* expression maintains a close positive relationship with *adiponectin* in adipose tissue. Thus, a strong positive relationship between ZAG and adiponectin gene expression in both VAT and SAT depots has been described [15], [16]. Administration of recombinant ZAG to human adipocytes can increase adiponectin release in mature cells [16]. In this line, a negative association has been described between ZAG expression in adipose tissue and insulin resistance measured by homeostasis model assessment (HOMA-IR) [15], [16]. In a recent study, administration of recombinant ZAG to hyperglycemic (ob/ob) mice produced a significant decrease in basal plasma insulin due to an increase in glucose consumption. This “hypoglycaemiant” effect of ZAG also seems to be mediated through stimulation of a β3 receptor [17].

In light of this supposed metabolic effect of ZAG and considering the close relationship observed with adiponectin we hypothesize that ZAG may be related with hydrocarbonate metabolism during pregnancy, mainly in GDM patients by influencing the insulin-resistance milieu. To test this hypothesis, we analyzed ZAG serum levels in a well-characterized cohort of pregnant women with GDM and normal glucose tolerance (NGT). Furthermore, we also analyzed the relationship between cord blood ZAG (cbZAG) levels and metabolic and anthropometric parameters of their offspring.

## Materials and Methods

A prospective analytic case-control study was conducted at the Joan XXIII University Hospital. Three hundred and seventy-seven pregnant Caucasian women were recruited at the time of antepartum screening for GDM. All of the women who participated underwent a 3-h, 100-g oral glucose tolerance test (OGTT) and were monitored from the time of inclusion until delivery. Serum and plasma samples were kept in a GDM biobank collection. The Joan XXIII Hospital Ethics Committee approved the study and written informed consent was obtained from all participants. Following the Spanish GDM guidelines and according to the OGTT, women with 2 or more values above the threshold proposed by the National Diabetes Data Group [18], [19] were considered GDM, and women who had all the values below the threshold were classified in the normal glucose tolerance (NGT) group. Women with only one value above the threshold after oral glucose tolerance were excluded from the study. In this study we included two hundred and seven pregnant women who fulfilled the following criteria at the end of pregnancy, 1) a singleton pregnancy, 2) accurate gestational age confirmed by an ultrasound examination before 20 weeks of gestation, 3) the absence of fetal abnormalities identified at birth, 4) cord blood sample was obtained at delivery, and 5) NGT or GDM diagnosed before 30 weeks of pregnancy. Women with only one value above the threshold after OGTT were excluded from the study. One hundred and sixty five women also fulfilled an additional criterion 6) neonatal biometry within the 48 hours of delivery.

GDM women were given a personalized diet with 40% of carbohydrates and they were instructed to self-monitor blood glucose 6 times a day (fasting and 1 hour postprandial) daily. Insulin therapy was recommended when fasting glucose values were repeatedly ≥95 mg/dl and or 1 hour postprandial values were >140 mg/dl. According to these criteria, 48 women were treated only with diet and 29 women required the addition of insulin.

Clinical and demographic data: Upon inclusion, demographic and historical information was collected via an interviewer-administered questionnaire focused in personal medical and obstetrical history, information regarding the current pregnancy with special attention to risk factors for gestational diabetesAlso, maternal anthropometric data including height, pre-pregnancy weight, and weight at the end of pregnancy were collected. Pre-pregnancy BMI was calculated using the formula: pre-pregnancy weight (kg)/(height (m))^2^. Increased BMI was calculated by the formula BMI gain = final BMI – pre-pregnancy BMI. Neonatal length and weight were determined in all participants using a measuring board to the nearest 0.1 cm and a calibrated scale to the nearest 10 g. In a subgroup of one hundred and sixty five offspring, more complete fetal anthropometry was also measured. Triceps, biceps, subescapular, and flank skinfold thickness were measured with Holtain skinfold callipers (Chasmors Ltd, London UK). The circumference of the head, chest, abdomen at the umbilicus, and upper and lower limbs was measured with a mid-upper arm tape (Re-usable Lasso-o, Harlow, UK). The length of the upper and lower limbs was measured with an anthropometer (Chasmors Ltd, London UK). Newborn body composition was estimated by the formula validated by Dauncey [20]. This method assumes that the trunk and the upper and lower limbs are cylinders covered with a layer of fat. The head is considered a sphere without a fat layer. The triceps skinfold is used to estimate the fat covering limbs whereas the subescapular skinfold estimates the fat thickness of the trunk. The volume of the subcutaneous layer of fat covering each cylinder is estimated by the formula: Length×circumference×the correspondence skinfold value. Total body fat is the sum of volumes that cover each cylinder (two upper limbs+two lower limbs+trunk)×0.9 (the density of human fat tissue). We first calculated fat mass (FM), and fat-free mass (FFM) was obtained by the formula = Birth weight-FM.

Laboratory measurements: The 100 g-OGTT was performed in the morning after overnight fasting. Venous blood samples were drawn at baseline and 60, 120 and 180 minutes following ingestion of standard 100-g glucose load. Venous blood from the umbilical cord was obtained immediately after delivery. Glucose levels were determined in an ADVIA 2400 (Siemens AG, Munich, Germany) autoanalyzer using the standard enzyme methods. Fasting plasma insulin and C-peptide were determined by immunoassay in an ADVIA Centaur System (Siemens AG, Munich, Germany). This assay shows a cross-reactivity of lower than 0.1% to intact human proinsulin and the primary circulating split form. HOMA-IR was determined according to the following equation: fasting plasma glucose (mmol/L)×fasting plasma insulin (µU/ml/22.5) [21].

Serum ZAG levels were measured by sandwich ELISA (Bio-Vendor Laboratory Medicine, Inc., Palackeho, Czech Republic). The intra and inter-assay CVs were lower than 5 and 6.6%, respectively and assay sensitivity was 0.673 ng/ml.

Serum adiponectin levels were determined using a human ELISA kit (multimeric Adiponectin ELISA Kit; Bühlmann, Schönenbuch, Switzerland). The intra and inter-assay CVs were lower than 15% and assay sensitivity was 0.08 ng/ml.

### Statistical analysis

Statistical analysis was performed by using the SPSS statistical package (version 13; SPSS, Chicago, IL). The 1-sample Kolmogorov-Smirnov test was performed to verify the normal distribution of the quantitative variables. For clinical and anthropometrical variables, normal distributed data are expressed as mean values (±SD), and for variables with a non Gaussian distribution, values are expressed as median (25–75^th^ percentile). Categorical variables were reported by number (percentages). Comparisons of quantitative variables between groups were performed by either Student's t-test or the Mann-Whitney U test according to the data distribution. Associations between quantitative variables were evaluated by Pearson correlation analysis or Spearman correlation for non-normally distributed variables. The independence of the associations of maternal ZAG (mZAG) and cbZAG with clinical and analytical variables was evaluated by stepwise multiple linear regression analysis. Variables with a significant association in the bivariate analysis or those known to be related with the physiopathology of insulin-resistance were included as independent ones in the model. All models were also adjusted for age. Statistical significance was accepted at the level of P<0.05.

## Results

One hundred and thirty NGT and seventy-seven GDM pregnant women and their respective offspring were included in the study.

### Maternal outcome

The main clinical and analytical variables of the study participants are summarized in table 1.

**Table 1:** Clinical and metabolic characteristics of the population studied.

	NGT(130)	GDM(77)	P
**Maternal characteristics**			
Age (years)	31.35±4.90	31.83±5.26	0.512
Gestational age (weeks)	27 (26–28.50)	28 (27–29)	0.252
Pre-pregnancy BMI (kg/m^2^)	23.23 (21.23–27.63)	24.72 (22.34–28.36)	0.083
Gain in BMI (kg/m^2^)	4.82±2.04	3.70±2.08	**<0.001**
SBP (mm Hg)	116.05±13.86	118.68±12.56	0.172
DBP (mm Hg)	68.09±9.73	68.0±8.77	0.993
Tobacco use n (%)	21 (16.15)	15 (19.48)	1.0
Insulin treated n (%)	-	29 (37.66)	-
Fasting glucose (mg/dl)	80.44±6.89	85.69±10.40	**<0.0001**
Fasting insulin (µIU/ml)	7.58 (5.82–13.0)	10.03 (7.11–15.27)	**0.007**
HOMA-IR	1.49 (1.11–2.53)	2.13 (1.43–3.58)	**0.002**
Cholesterol (mmol/liter)	6.68±1.10	6.59±1.09	0.567
HDL cholesterol (mmol/liter)	1.91±0.31	1.85±0.34	0.219
Triglycerides (mmol/liter)	1.88 (1.52–2.27)	1.99 (1.64–2.56)	0.80
mZAG (µg/ml)	46.88±9.36	47.25±11.74	0.818
mAdiponectin (µg/ml)	5.80±2.37	4.90±2.13	**0.006**
**Neonatal characteristics**			
Gestational age at delivery	39 (38–40)	39 (38–40)	0.560
Male sex n (%)	60 (46.1)	41 (53.2)	0.325
Birth weight (g)	3278.07±477.53	3244.22±464.47	0.617
cbInsulin (µIU/ml)	4.33 (2.46–6.89)	5.36 (2.98–9.91)	**0.045**
cbZAG (µg/ml)	20.67±7.15	20.32±6.31	0.713
cbAdiponectin (µg/ml)	17.62±6.34	17.04±6.72	0.548
FM (n) (g)	(97) 318.38±132.64	(68) 291.38±131.40	0.198
FFM (n) (g)	(97) 2794.60±369.63	(68) 2778.35±358.16	0.750

Value data are presented as mean ± SD or median (25–75th percentile) for nonnormally distributed.

variables. mAdiponectin: maternal adiponectin, cbAdiponectin: cord blood adiponectin, SBP: systolic.

blood pressure, DBP: diastolic blood pressure.

As expected GDM women had higher levels of fasting glucose, fasting insulin and HOMA-IR. By contrast NGT women showed a higher gain in BMI during pregnancy (4.82±2.04 *vs.* 3.70±2.08 kg/m^2^ P<0.001). The remaining variables did not show any differences between the groups. Serum mZAG circulating levels were similar both in NGT and GDM women. Furthermore, mZAG concentrations were similar in insulin or in diet-treated GDM women (45.88±11.59 µg/ml *vs.* 48.01±11.98 µg/ml respectively).

We also analyzed the influence of pre-pregnancy weight in ZAG levels. No differences in mZAG levels were observed between lean (BMI≤25 Kg/m2) and overweight (BMI>25 Kg/m2) women, either when we considered the whole group (47.91±10.67 µg/ml *vs.* 45.76±9.63 µg/ml respectively) or when NGT (47.63±9.57 µg/ml *vs.* 46.15±8.45 µg/ml) and GDM groups (48.46±12.66 µg/ml *vs.* 45.24±12.66 µg/ml) were analyzed separately.

### Fetal outcome

The main clinical and analytical variables of the offspring are summarized in table 1.

Cord blood insulin levels were higher in offspring of GDM when compared with NGT pregnant women. Gender distribution, mean birth weight (BW) and gestational age at delivery were similar in both groups. There were no significant differences in cord blood ZAG (cbZAG) levels between GDM and NGT groups and no effect on cbZAG levels regarding GDM treatment (insulin or diet only) was observed (21.79±6.74 µg/ml *vs.* 19.63 µg/ml±5.83 respectively).

When we analyzed the relationship between cbZAG and mZAG levels in the whole group we observed that cbZAG concentrations were more than twofold lower than mZAG levels (20.54±6.84 µg/ml *vs.* 47.02±10.28 µg/ml respectively P<0.001). Similar results were obtained when we studied the GDM (20.32±6.31 µg/ml *vs.* 47.25±11.74 µg/ml P<0.001) and NGT groups (20.67±7.15 µg/ml *vs.* 46.88±9.37 µg/ml P<0.001) separately.

### Relationship between ZAG levels with clinical and analytical variables

**Figure 1:**
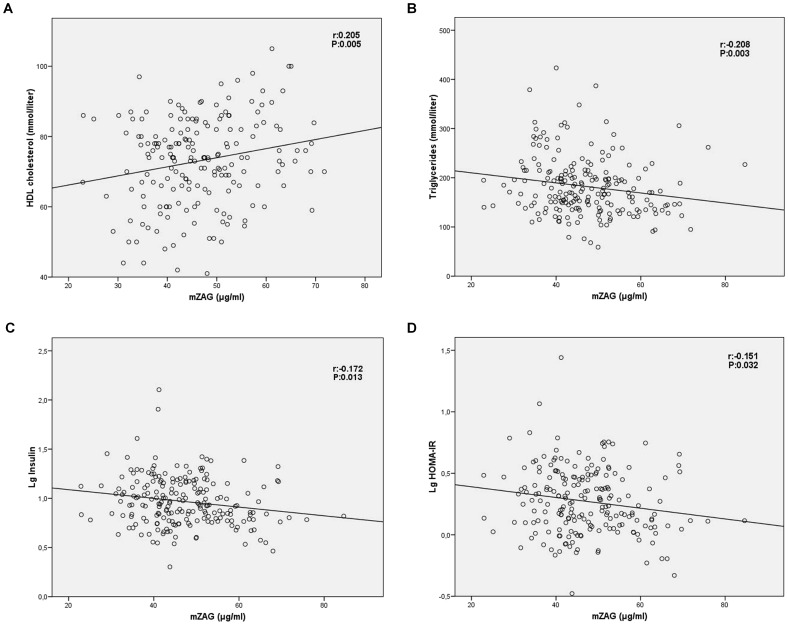
Correlation between serum mZAG levels and HDL cholesterol levels (A), triglyceride levels (B), Insulin logarithm (Lg Insulin) (C) and HOMA-IR logarithm (Lg HOMA-IR) (D).

In the bivariate correlation analysis, no association between mZAG and cbZAG levels was observed. Serum mZAG concentrations showed a positive correlation with HDL cholesterol levels and a negative correlation with triglyceride levels, insulin and HOMA-IR (figure 1). No correlation between mZAG concentrations and fetal variables were observed.

**Figure 2:**
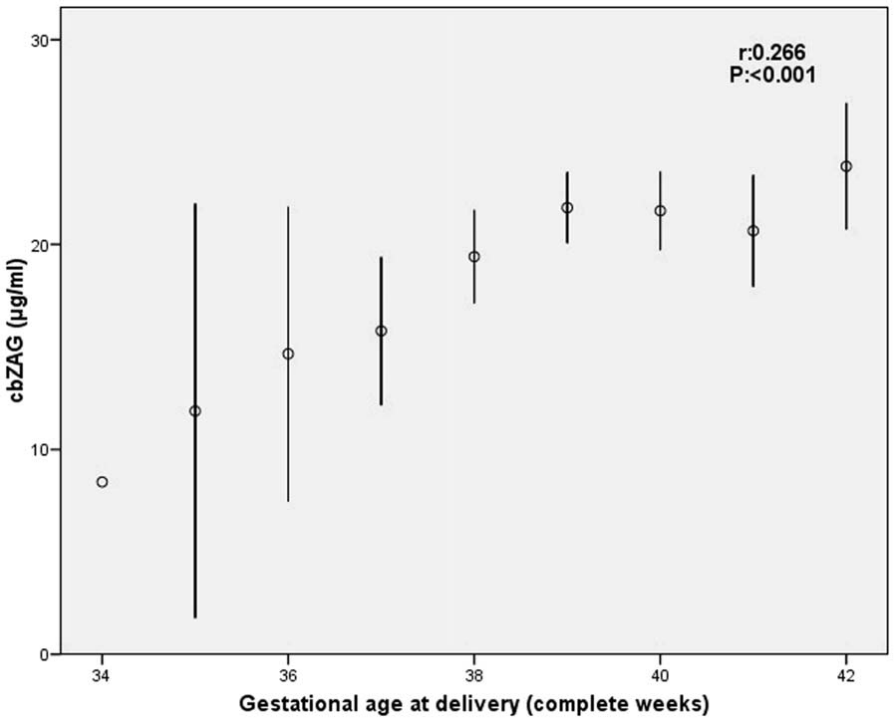
Relationship between serum cbZAG levels and gestational age at delivery.

**Figure 3:**
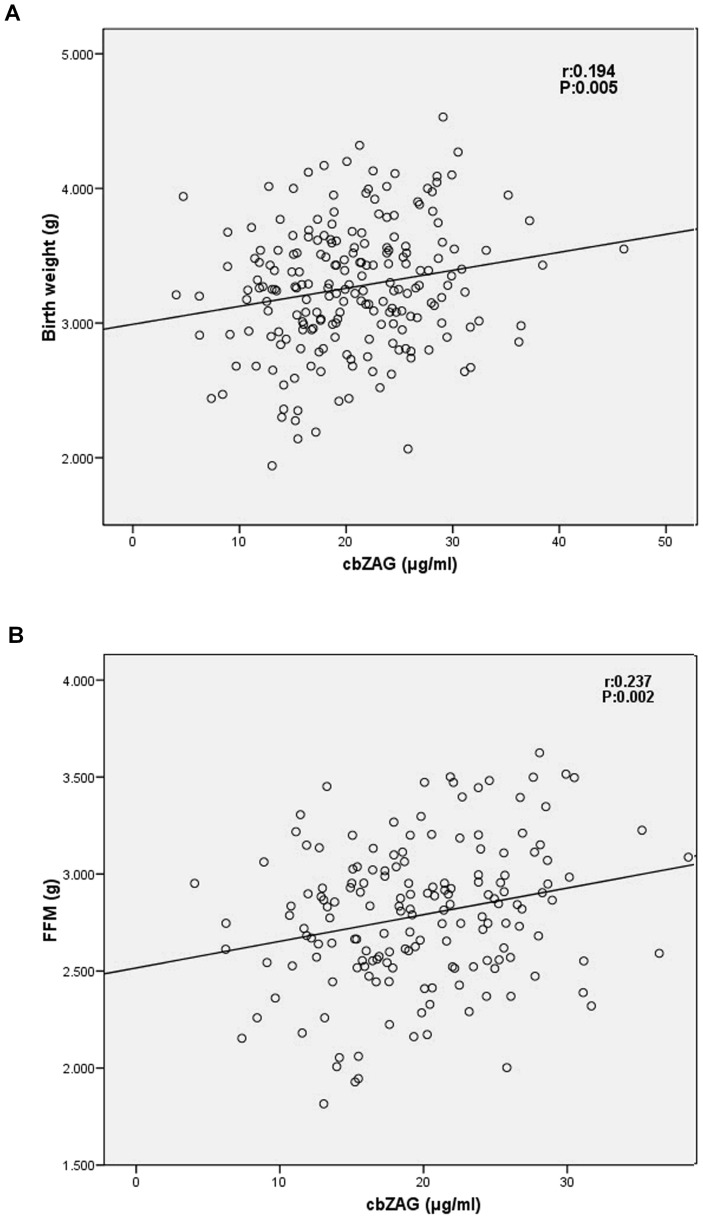
Correlation between serum cbZAG levels and anthropometric variables: birth weight (A) and fat-free mass (FFM) (B).

Serum cbZAG levels were positively associated with gestational age at delivery (figure 2), BW and FFM (figure 3). There were no correlations between cbZAG levels and maternal variables.

There was a positive correlation between mZAG and maternal adiponectin levels (r = 0.205; P = 0.003). Concerning cbZAG levels, no relationship with maternal or cord blood adiponectin levels was observed.

To strengthen the independence of these relationships, a series of stepwise multiple linear regression analysis models was constructed:

First model with mZAG as dependent variable; we include HDL-cholesterol, triglycerides, HOMA-IR, cbZAG, maternal adiponectin, GDM, maternal age and pregravid BMI as independent variables. HDL cholesterol emerged as the sole positive determinant of mZAG (B = 0.150; P = 0.039).

Second model with cbZAG as dependent variable; we include gestational age at delivery, BW, FFM, mZAG, cord blood adiponectin, GDM and neonatal sex as independent variables. The final regression model identified gestational age at delivery as the sole and potent positive predictor (B = 0.292; P = <0.0001).

Third model with maternal adiponectin as dependent variable; we include mZAG, HOMA-IR, pre-pregnancy BMI, maternal age and gain in BMI as independent variables. The result showed that maternal adiponectin was mainly predicted by mZAG (B = 0.187; P = 0.007) and HOMA-IR (B = −0.181; P = 0.009).

## Discussion

Insulin resistance plays a pivotal role for the proper maintenance of metabolic homeostasis during pregnancy. The secretory function of adipose tissue has gained attention in the adequate functioning of glucose metabolism when insulin resistance becomes evident, as occurs in the last months of pregnancy. However, human data are very scarce regarding adipokines during pregnancy. In this study we have described for the first time that the serum ZAG levels during pregnancy are associated with a healthier metabolic profile, with independence of the glucose metabolism. Furthermore, the absence of relationship with pre-pregnancy BMI suggests that adipose tissue is not a major determinant of its circulating levels during pregnancy, although we cannot discard a local metabolic role of ZAG.

ZAG is a relatively new player in metabolic disturbances of insulin resistance-derived diseases, but the previous studies in non-pregnant subjects have yielded heterogeneous results. Thus, in obesity, low [14], [22], unchanged [15] or increased [23], [24] circulating levels have been reported. Furthermore, in metabolic syndrome and type 2 diabetes similar results have been observed, with increased or comparable levels to healthy counterparts [23]–[25]. Differences in the ethnic backgrounds and in patient characteristics might explain these inconsistent results.

Only one previous report exists analyzing ZAG levels in pregnancy in women with preeclampsia. In this report, high levels of serum ZAG were described in preeclampsia, but no clear association with traits of metabolic syndrome were detected [26]. By contrast, in our cohort, circulating maternal ZAG levels were associated with a better metabolic profile including a weak positive association with maternal adiponectin. These discrepancies may be due, in part, to the greater number of women included in our study. Nevertheless, HDL cholesterol remained the unique determinant of mZAG levels after controlling for confounding factors, toning down the usefulness of this protein in maternal serum during pregnancy as a biomarker for a worse metabolic profile. In this line, pregnant women with altered glucose metabolism showed similar mZAG circulating levels to NGT women, despite the inverse association with the insulin resistance parameters (insulin, HOMA-IR index and triglycerides) detected in the univariate analysis. Interestingly, when we analyze the variables influencing circulating adiponectin levels in our cohort, mZAG was revealed as one of the main predictors (positively associated) along with HOMA-IR (negatively associated). Thus, we cannot exclude an influence of ZAG on glucose metabolism, at least indirectly, by influencing other hormones with a more direct role in the generation of insulin-resistance such as adiponectin.

Another new finding reported herein is the low levels of ZAG in cord blood when compared with mZAG levels. We are aware of the limitations of the study design regarding no mechanistic interpretations. However, the positive associations observed between cbZAG and several anthropometric markers measured in the newborn lead us to speculate as to a different role of this cytokine in the fetal environment. On the other hand, the lower ZAG levels in offspring may protect the fetus from excessive lipolytic activity during the latter part of pregnancy when the fetal anabolic functions dominate, unlike adiponectin, which stimulates adipogenesis in experimental models [27]. In fact, the main determinant of cbZAG levels was maternal gestational age at delivery; the higher the age, the higher cbZAG levels, suggesting that umbilical ZAG is determined by the maturity of adipose tissue rather than the quantity of fat mass. We cannot rule out an influence of circulating mZAG at the end of pregnancy on cbZAG levels at delivery. However, the high molecular weight of ZAG makes it difficult for this protein to be transferred across the placenta influencing cbZAG levels. In addition, ZAG is one of the 136 different proteins detected in the proteomic analysis of normal human amniotic fluid and it is secreted from the fetal liver indicating a physiological role in the wellbeing of the fetus during normal pregnancy [28] without discarding a placental implication in its metabolism.

In summary, we have observed higher ZAG concentrations in pregnant women than in the cord blood of their offspring, and cord blood ZAG concentrations were determined by the gestational age. Despite no differences being observed in ZAG concentrations between GDM and NGT women, maternal ZAG is associated with a better metabolic profile in pregnant women, as shown by the positive association with HDL cholesterol and adiponectin concentrations.
